# Characterization of the immune cell infiltration landscape in myxofibrosarcoma to aid immunotherapy

**DOI:** 10.3389/fimmu.2022.916915

**Published:** 2022-07-22

**Authors:** Zi-Yue Zhao, Zhuo-Yuan Chen, Bin Yu, Bo Xiao, Li-Yan Liu, Yu Xia, Ao-Yu Li, Ping-Xiao Wang, Cheng Xiang, Chao Liu, Hui-Qin Yang, Hui Li, Tao Xiao

**Affiliations:** ^1^ Department of Orthopedics, Second Xiangya Hospital, Central South University, Changsha, China; ^2^ Orthopedic Biomedical Materials Engineering Laboratory of Hunan Province, Changsha, China; ^3^ Department of Orthopedics, The Affiliated yanan Hospital of Kunming Medical University, Kunming, China

**Keywords:** MFS, ICI, TME, prognosis, immunotherapy

## Abstract

Myxofibrosarcoma (MFS) is a highly malignant subtype of soft tissue sarcoma, accounting for 5% of cases. Immunotherapy guided by immune cell infiltration (ICI) is reportedly a promising treatment strategy. Here, MFS samples (n = 104) from two independent databases were classified as ICI clusters A/B/C and gene clusters A/B/C. Then, a close relationship between ICI and gene clusters was established. We found that the features of these clusters were consistent with the characteristics of immune-inflamed tumors (cluster C), immune-desert tumors (cluster B), and immune-excluded tumors (cluster A). Moreover, cluster C was sensitive to immunotherapy. Finally, an independent ICI score was established to predict the therapeutic effect, which has prospects for application in guiding immunotherapy during clinical practice.

## Introduction

Myxofibrosarcoma (MFS) is an important subtype of soft tissue sarcoma, accounting for 5% of soft tissue sarcomas and predominantly occurring in the limbs of elderly men (1, 2). The pathological feature of MFS is pleomorphic tumor cells exhibiting nodular morphology in the myxoid stroma with an infiltrative growth pattern (3–5). Recently, surgery has become the mainstay of treatment for MFS (6). In low-grade MFS, the infiltration and growth characteristics lead to deceptive tumor tissue boundaries (highly malignant but with a low cellular appearance). The risk of metastasis is inevitable with high-grade MFS malignancy. Regrettably, the rate of *in situ* recurrence after the MFS resection remains high (7). Consequently, although the overall prognosis of MFS is better than that of soft tissue sarcoma, given its unique pathological characteristics, individualized and accurate treatment is still helpful in improving the prognosis and survival expectation (8). Accordingly, it is essential to construct a novel immune cell infiltration (ICI) prognosis signature for predicting the prognosis and guiding the personalized treatment of MFS patients.

It has been established that immune cell infiltration is an important feature of the tumor microenvironment (TME) (9). Research on ICI is essential for researchers to better understand the TME, with mounting evidence that the efficacy of immunotherapy can be improved by increasing the degree of ICI in TME (10). Indeed, research on ICI in tumor tissue undoubtedly contributes to developing treatment plans. Immune checkpoint inhibitors (ICIs) represent a relatively new treatment scheme and have been the subject of a series of studies to assess their efficacy in various tumors (11–13), with satisfactory results being achieved in the clinic (14, 15).

Compared with traditional treatments, ICIs play a crucial role in inducing a long-term immune response (16). In particular, researchers have conducted clinical experiments to evaluate the efficacy of pembrolizumab, nivolumab (anti-PD-1) and ipilimumab (anti-CTLA) alone or in combination for sarcoma treatment (17). A study showed that after immunotyping of soft tissue sarcoma according to the composition of TME, there were B-cell-rich tertiary lymphoid structures in the two subtypes of the immune-high group, which showed a high response rate to PD-1 blockade with pembrolizumab in a phase 2 clinical trial (18). A study showed that although PD-L1 can predict the clinical outcome of pazopanib (a type of tyrosine kinase inhibitor, TKI) for treatingsoft tissue sarcoma, predictive models are still warranted to determine which patient population will benefit from pazopanib (19).

A recent retrospective study of ICIs in sarcoma treatment by You et al. analyzed the treatment-related indicators of 61 sarcoma patients treated with ICIs. It was suggested that alveolar soft part sarcoma (ASPS), undifferentiated pleomorphic sarcoma (UPS), and myxofibrosarcoma (MFS) were sensitive to immunotherapy (20). Notwithstanding that ICIs represent a new type of immunotherapy different from traditional anti-tumor therapy, the drug toxicity and efficacy remain relatively unknown during treatment, and prediction methods to investigate the treatment reaction are quite limited; it can be challenging to determine and optimize the pseudo-progression (PP) and hyper-progression (HP) of ICI treatment in time (21). For the immunotherapy of various tumors, although monotherapy with ICIs yields a good prognosis, it is widely thought that the combination of ICIs and other targeted drugs yields a better curative effect. Accordingly, the combination of drugs has gradually become a new direction for immunotherapy: A study analyzed the data of 1,769 cases of metabolic renal cell carcinoma (MRCC) routinely collected in randomized controlled trials and found that ICIs combined with TKIs significantly improved the prognosis of MRCC patients (22). In a retrospective study on the prognosis of immunotherapy with ICIs for hepatocellular carcinoma (HCC), the researchers found that the identification of predictive biomarkers of response (such as TMB and PD-L1) could effectively help patients during immunotherapy, suggesting that the targeted study of prognostic biomarkers of immunotherapy has broad prospects (23). Therefore, our study substantiates that the prognosis prediction index of immunotherapy for MFS based on ICI score has clinical significance for guiding the optimization of immunotherapy.

## Methods

### Myxofibrosarcoma data collection

Clinical information and transcriptomic data of MFS patients were obtained from The Cancer Genome Atlas (TCGA) and Gene Expression Omnibus (GEO) databases. The TCGA-SARC was designated the “CASE” type by the TCGA, and the data were downloaded in fragments per kilobase per million (FPKM) format. The MFS survival data were searched in GEO, and the data which met the research requirements (n ≥30) were selected and downloaded based on the integrity of survival information and the sample size of the dataset. There were 40 samples from the TCGA and 64 samples from GEO (Dataset GSE 72545).

### Consensus clustering for the landscape of immune cell infiltration

To quantify the degree of infiltration of 22 different immune cell subsets in MFS tissue samples, the R package “CIBERSORT” was used to conduct immune cell infiltration typing and the most appropriate grouping number was selected for follow-up research. To ensure the stability of the classification, 1,000 iterations were performed. The immune/stromal cell content of every sample was assessed by the ESTIMATE algorithm to determine the purity of tumor samples. Additionally, the unsupervised clustering “PAM” method was used in the analysis based on EUCLIDEAN and WARD’s linkage. To explore the possible relationship between MFS-related genes and the ICI pattern at the genetic level, the differentially expressed genes (DEGs) involved in MFS samples were classified by the same method.

### ICI-related DEGs enrichment analysis and establishment of the ICI score

After genotyping MFS patients through unsupervised clustering, the “Boruta” algorithm and “PCA” were used to translate each sample and obtain various scores for the main characteristics. The difference between the ICI scores of the two main marker genes of each sample was the exact ICI score of this scheme: ICI score = ∑ PCI_A_ − ∑ PCI_B_. Then, Gene Set Enrichment Analysis (GSEA) enrichment analysis of the related pathways of the ICI high group and ICI low group was conducted. The ICI-related genes (DEGs) were classified by the “limma” R package according to the ICI in MFS samples. The appropriate number of genotypes was calculated according to the previous results to further explore the pattern of ICI. After preliminary processing, the data were corrected, and significant DEGs were screened based on the criteria: p <0.05 and absolute fold change >1. Then, Gene Ontology (GO) functional enrichment analysis was conducted.

### Independent verification of ICI score

MFS-related somatic mutation data were obtained from the TCGA. It is well-established that TMB is the number of mutations in the coding region of an exome (number of mutations detected in exon/mb length) (24). TMB is a validated scoring criterion for predicting tumor immunotherapy. Therefore, we used TMB as the standard to conduct differential expression analysis on MFS samples to confirm the sample composition, and then used the combination of ICI score and TMB score to conduct a stratified test to verify the independence of the ICI score.

### Data statistics and visualization

The Wilcoxon test analyzed the difference between the two groups, while the Kruskal–Wallis test was used for more than two groups. Kaplan–Meier survival curves were generated. Various R packages were used to visualize the results, including: “limma, e1071, estimate, corrplot, ConsensusClusterPlus, survival, survminer, pheatmap, reshape2, ggpubr, ggplot2, and Boruta.” Violin plots were generated by an online tool (http://vip.sangerbox.com/login.html).

## Results

### The immune cell infiltration landscape in the TME of MFS

**Figure 1:**
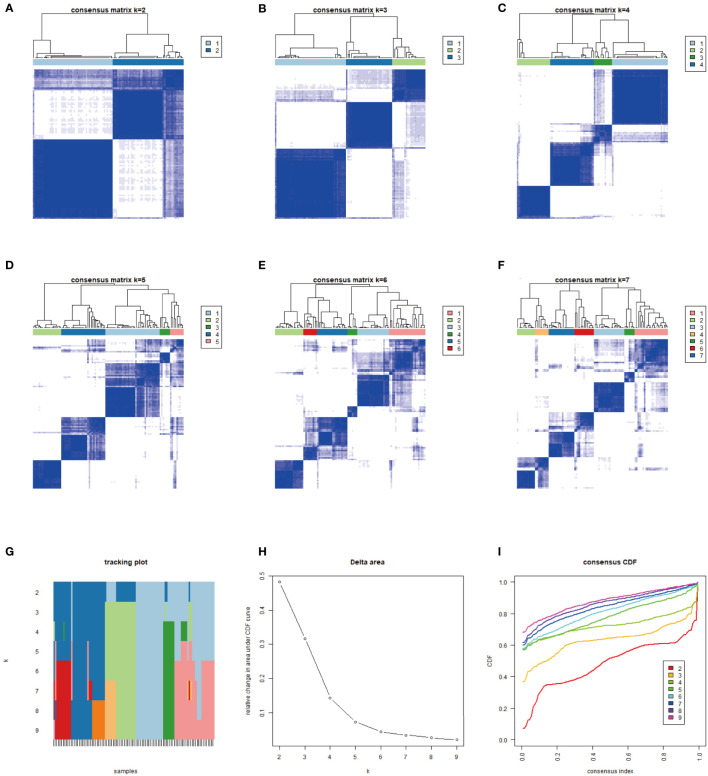
We typed 104 samples from the TCGA-MFS and GEO (GSM 72545) through CIBERSORT analysis. The samples were finally divided into 3 independent subtypes according to the stability of typing results. **(A–F)** represent the sample purity when the typing was 2–7: The blue square in the figure represents different classification aggregations. The darker the color and the smaller the number of blanks, the lower the difference in the aggregated samples and the higher the purity of typing. **(G–I)** reflect the purity of typing and the stability in the samples: **(G)** The abscissa is the sample, and the ordinate is the different classification, reflecting the stability between samples after different classification; **(H)** Cumulative Distribution Function (CDF) curve showing the sampling error in different classifications; **(I)** Explanation of CDF curve, although the results of both analyses were better, we still chose 3 types in combination with **(A–F)**.

**Figure 2:**
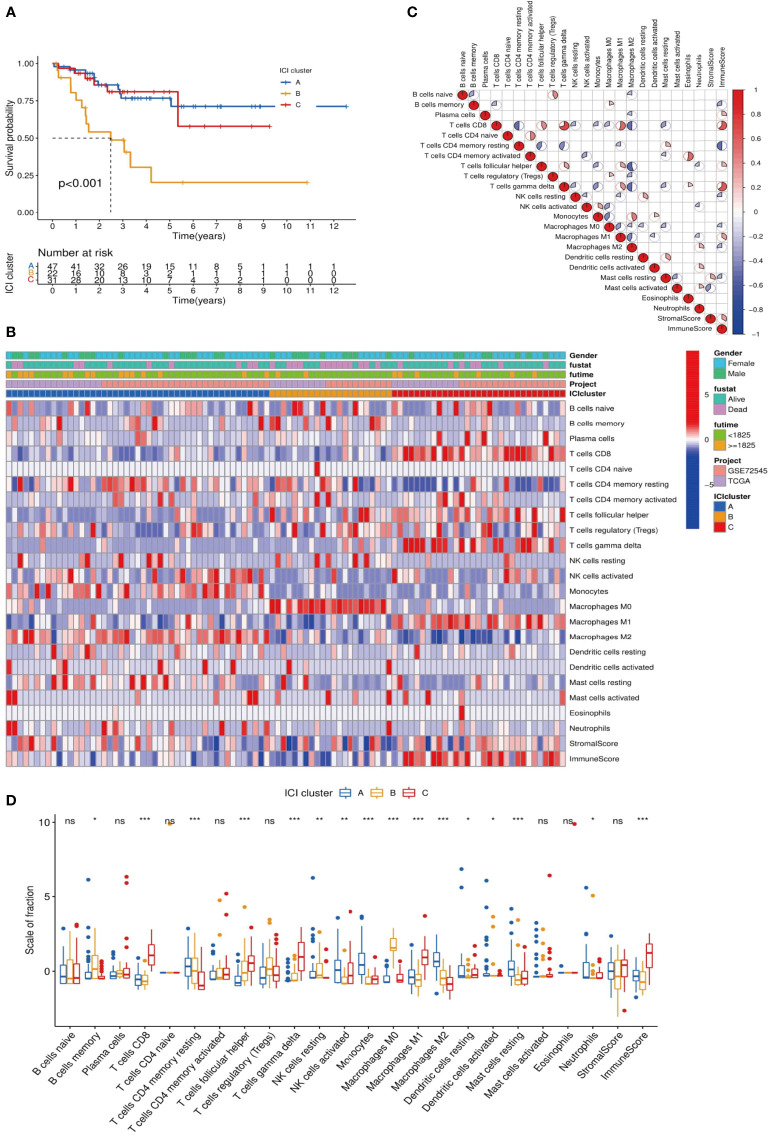
Analysis of differences among ICI subtypes and immune infiltration characteristics. **(A)** We analyzed the difference in Overall Survival (OS) among the 3 ICI subtypes and visualized the details *via* a K-M survival curve: compared with ICI cluster A and ICI cluster C, the OS of ICI cluster B was significantly lower, p <0.001; **(B)** Unsupervised cluster analysis was used to analyze the distribution of immune infiltration characteristics in MFS samples. The abscissa represents the immune infiltrating characteristics, and the ordinate represents independent samples; **(C)** We explored the relationships among 24 immune infiltrating characteristics (22 kinds of immune infiltrating cells and Stromal/Immune Score): red indicated a positive correlation, and blue indicated a negative correlation. The higher the correlation, the larger the pie chart area; **(D)** The differences in expression of 24 immune infiltration characteristics in 3 ICI subtypes are visualized in a box plot: ***p <0.001, **p <0.01, and *p <0.05 ns p>0.05,no significance.

CIBERSORT and ESTIMATE algorithms were used to analyze the MFS tumor tissue samples and quantify immune cells in MFS. The 104 tumor samples of MFS from the TCGA and GEO were divided into three subtypes by unsupervised clustering according to the pattern of ICI and the stability of the results (**Figure 1**). After cluster analysis, 100 of 104 samples with meaningful data were retained and classified as follows: ICI cluster A (n = 47), ICI cluster B (n = 22), and ICI cluster C (n = 31). There were significant differences in the prognosis and outcome among the three ICI types (log-rank test, p <0.001). We found that the overall survival (OS) of ICI cluster B was significantly lower than the other two sub-types (**Figure 2A**). As seen in the heatmap, significant differences in clinical characteristics were found among the three ICI clusters, while the box plot showed the differences in expression of 22 immune cell subtypes and the Stromal/Immune Score. Significantly higher infiltration levels of resting memory CD4 T cells (p <0.001), activated NK cells (p <0.01), monocytes (p <0.001), M2 macrophages (p <0.001), and resting mast cells (p <0.001) were found in ICI cluster A compared with the other 2 clusters. Memory B cells (p <0.05), resting NK cells (p <0.01), and M0 macrophages (p <0.001) exhibited significantly higher infiltration levels in ICI cluster B. Finally, CD8 T cells (p <0.001), follicular helper T cells (p <0.001), gamma delta T cells (p <0.001), and M1 macrophages (p <0.001) exhibited higher infiltration levels in ICI cluster C, with the higher results in the Stromal/Immune Score (**Figure 2B**). We used a correlation coefficient matrix heat map to show the interaction among the immune infiltration characteristics. A negative correlation was found between M2 macrophage and follicular helper T cells, M2 macrophage and CD8 T cells, M2 macrophage and gamma delta T cells, resting memory CD4 and CD8 T cells, and immune score and resting memory CD4 T cells. A positive correlation was found between gamma delta and CD8 T cells, Immune Score and CD8 T cells, Immune Score and gamma delta T cells, eosinophils and activated CD4 memory T cells, and M1 macrophage and CD8 T cells (**Figures 2C, D**).

**Figure 3:**
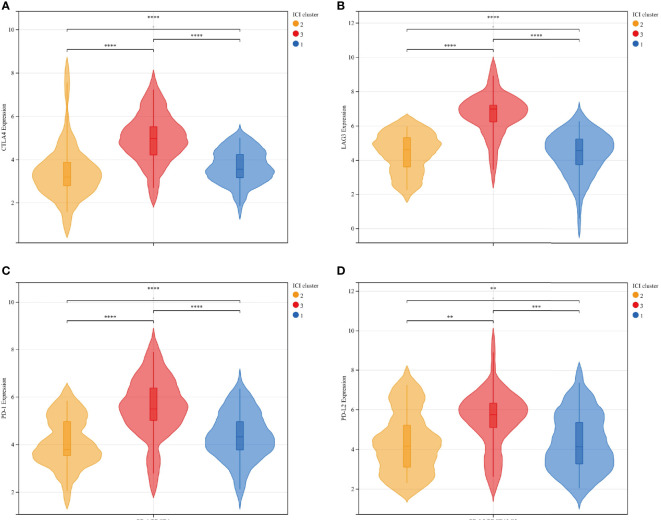
The expression differences between immune checkpoint-related genes and ICI subtypes were represented by a violin plot: CTLA4 **(A)**, LAG3 **(B)**, PD-1 **(C)**, and PD-L2 **(D)**: There was a significant statistical difference among the three groups. Among the four independent genes, ICI cluster C exhibited significantly higher expression than ICI clusters A and B, and the average expression of ICI cluster B was relatively lower than ICI cluster **(A)** ICI clusters: 1-blue-A, 2-yellow-B, 3-red-C. ****p<0.0001, ***p <0.001, **p <0.01, and *p <0.05.

To further explore the feasibility of immunotherapy in MFS, the expression differences in common immune checkpoint-related genes in ICI typing were reflected by a violin plot, in which CTLA4, LAG3, PD-1, and PD-L2 were significantly different in three ICI sub-types (p <0.01). CTLA4, LAG3, PD-1, and PD-L2 expression in ICI cluster C was significantly higher than in clusters A and B, and there were significant differences among the three ICI subtypes of these four genes (**Figure 3**).

### Genotyping and difference analysis of ICI-related genes

**Figure 4:**
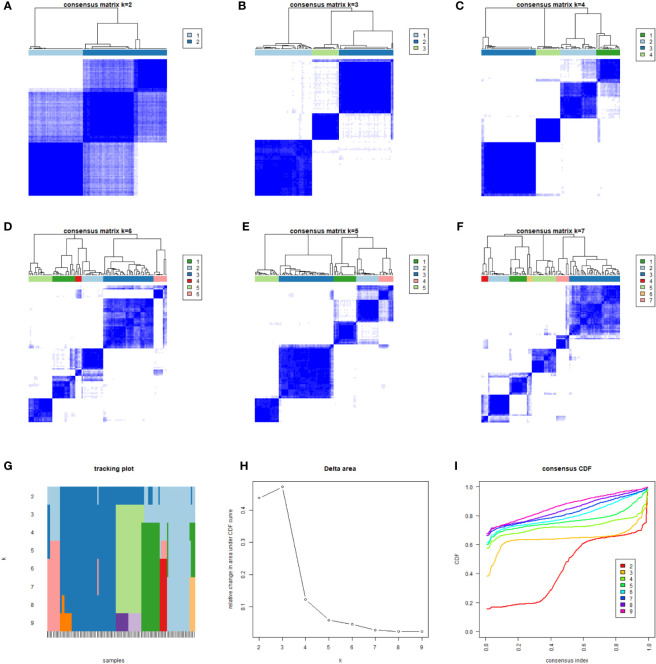
We classified 104 different samples through unsupervised clustering. According to the obtained correlation results among types **(A–F)**, the number of gene types was set to 3. The correlation among types was comparable; a low correlation was associated with stable classification results **(G–I)**.

The gene expression of all samples was analyzed by ICI typing and the R package “limma.” After three repetitions, 689 DEGs were obtained under the conditions of |logFC |>1 and corrected p-value <0.05. By clustering the obtained DEGs of the samples with the same arithmetic as the ICI subtypes, the sample sub-types according to genes could also be calculated, which were called gene clusters. Considering the stability within the sub-types, three gene sub-types were divided from the differentiated samples (n = 104): gene cluster A (n = 43), gene cluster B (n = 41), and gene cluster C (n = 20). Gene clusters corresponded to ICI clusters (ICI cluster A–gene cluster A, ICI cluster B–gene cluster B, ICI cluster C–gene cluster C). In the prognostic K–M curve related to genotyping, the OS of gene cluster A was significantly lower than that of the other two clusters (p <0.001) (**Figure 4**).

**Figure 5:**
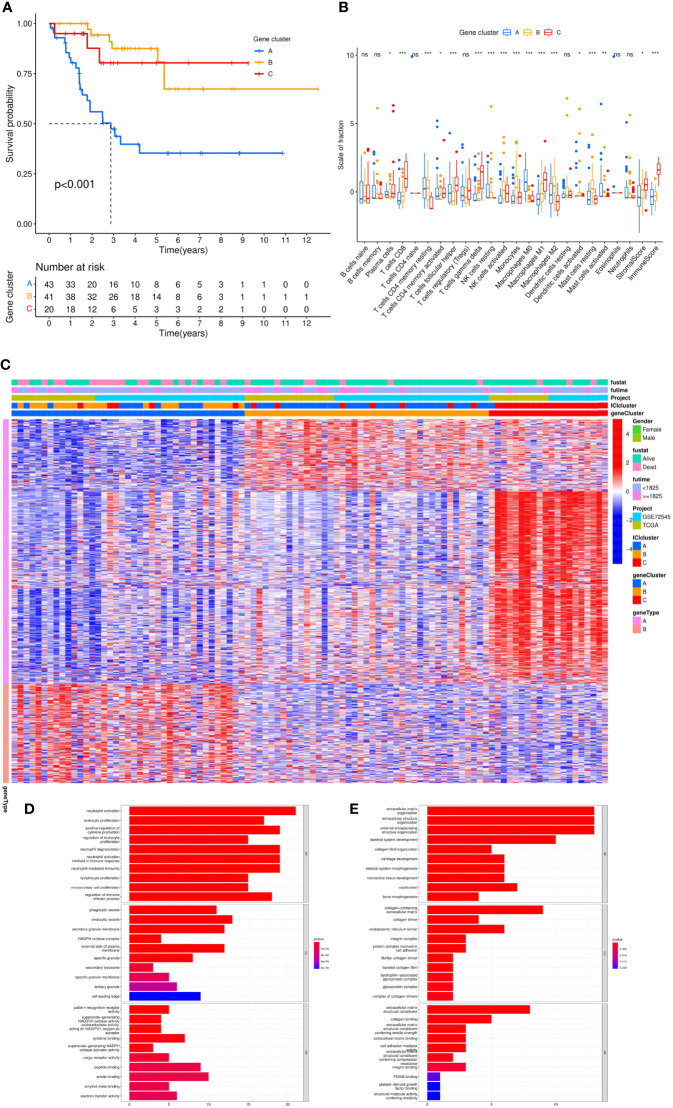
Differential analysis of genotyping of immune infiltration. **(A)** After unsupervised cluster analysis of DEGs and samples, we divided the samples into three independent gene clusters, the overall survival (OS) was analyzed by Kaplan–Meier analysis, and the log-rank test showed that P <0.001. **(B)** The differences in expression among 24 kinds of immune infiltrating characteristics in the 3 gene clusters were visualized in a box plot and statistically analyzed by Kruskal–Wallis test. **(C)** The clinical information was divided into two types. The abscissa was the samples, and the ordinate was the genes. **(D, E)** According to gene type A and gene type B, which were positively correlated with the ICI model in DEGs, the ordinate was the name of GO, the abscissa was the number of enriched genes, and the color represented the significance of the correlation (red indicated a positive correlation and blue indicated a negative correlation). ***p <0.001, **p <0.01, *p <0.05 and ns p>0.05,no significance.

KM analysis of three independent gene clusters showed significant differences in OS. The OS of gene cluster A was significantly lower than that of the other two clusters (log-rank test, p <0.001) (**Figure 5A**). To further clarify the difference in ICI characteristics in the genotyping of MFS, the expression of 24 immune infiltrating characteristics in 101 differentiated MFS samples was analyzed by genotyping. M0 macrophage (p <0.001) exhibited high infiltration levels in gene cluster A; activated NK cells (p <0.001), monocytes (p <0.001), M2 macrophages (p <0.001), activated dendritic cells (p <0.05), and resting mast cells (p <0.001) were highly expressed in gene cluster B. Plasma cells (p <0.05), CD8 T cells (p <0.001), activated CD4 memory T cells (p <0.05), follicular helper T cells (p <0.001), gamma delta T cells (p <0.001), M macrophages (p <0.001), Stromal Score (p <0.05), and Immune Score (p <0.001) were significantly overexpressed in gene cluster C. Additionally, the expression of the resting CD4 memory of T cells in gene cluster C was lower than in gene clusters A and B (**Figure 5B**).

Subsequently, we sought to stratify according to the relationship between gene expression and ICI characteristics of MFS samples. Positive correlated genes were attributed to class A (n = 70) while negative correlated genes to class B (n = 29), which was displayed in a heatmap (**Figure 5C**). To describe the relationship between these genes and biological processes (BPs), cellular components (CCs), molecular function (MF), and GO enrichment analysis were conducted. In class A, genes were involved in the proliferation and activation of immune cells, including the activation and degranulation of neutrophils, the proliferation and regulation of leukocytes, positive regulation of cytokine production, the proliferation of lymphocyte and mononuclear cells, which mostly occurred in secretory vesicles, NAD(P)H oxidase complex, and secondary lysosomes in the plasma membrane. Potential molecular functions include the activity of pattern recognition receptors, superoxide-generating NAD(P)H oxidase and oxidoreductase, and the binding of cytokine, peptide, amide and amyloid-beta (**Figure 5D**). Moreover, we found that class B had a close relationship with the composition of extracellular matrix and was significantly enriched in the biological function of extracellular matrix components of tumor tissue by participating in the composition of extracellular matrix in collagen, collagen trimer, endoplasmic reticulum cavity, integrin complex, and protein complex involved in cell adhesion. (**Figure 5E**).

**Figure 6:**
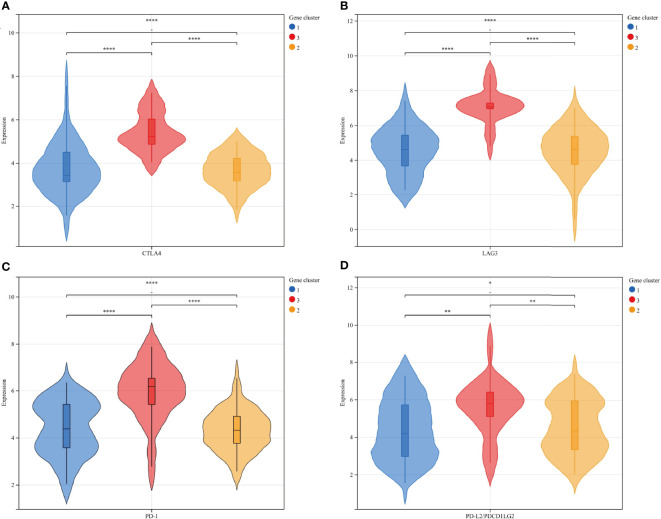
The expression differences of 4 immune checkpoint-related genes in gene subtypes were consistent with ICI subtypes: CTLA4 **(A)**, Lag3 **(B)**, PD-1 **(C)**, and PD-L2 **(D)**: Gene clusters: 1-blue-A, 2-yellow-B, 3-red-C. **** p<0.0001 ***P <0.001, **P <0.01, and *P <0.05.

To verify the consistency between ICI typing and genotyping, we analyzed the differences in four immune checkpoint-related genes with significant expression differences in ICI subtypes by genotyping. Similar to ICI typing, the four differential genes (CTLA4 (A), Lag3 (B), PD-1 (C), and PD-L2 (D)) in gene cluster C were significantly higher than in the other two clusters. Statistical differences among the three subtypes were also significant. The consistency of the expression of immune checkpoint genes in different unsupervised cluster typing confirmed the rationality of ICI typing in MFS (**Figure 6**).

### Acquisition of ICI model score and verification with tumor mutation burden

**Figure 7:**
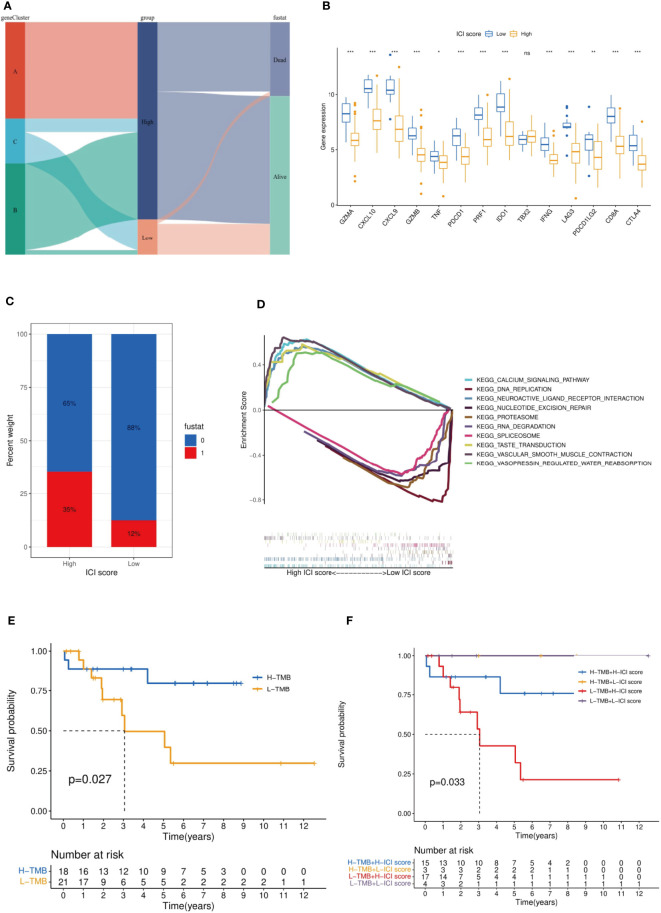
Established and verified the ICI score. **(A)** The relationship among gene clusters, ICI high or low groups and survival outcomes is visualized in a Sankey diagram. **(B)** Based on the ICI score, we analyzed the expression difference among the immune checkpoint genes and immune-activating genes. It should be noted that PDCD1LG2 is another name for PD-L2. **(C)** Effect of ICI score on patient survival. **(D)** GSEA indicated significantly enriched signaling pathways corresponding to high and low ICI score groups. **(E)** Survival analysis was performed by TMB score in our selected MFS samples. **(F)** MFS samples were stratified by TMB score and the ICI score established in this study. ***p<0.001,**p<0.01,*p<0.05 and ns p>0.05, no significance.

The rationality and stability of the ICI model were determined in advance. The feature genes and the related sample expression volumes were extracted according to the ICI classification using the “Boruta” algorithm. Then, the “PCA” algorithm was used to obtain the ICI score. High (n = 88) and Low (n = 16) groups were obtained from the samples according to their source, gene clusters, and clinical information. The Sankey diagram provided an objective overview of the relationship among gene clusters, survival outcomes, and ICI scores. All the genes of cluster A and most genes of cluster B belonged to the ICI High group, and the remaining genes of cluster B and some of cluster C belonged to the ICI Low group. Compared with the ICI High group, the ICI Low group reflected a high probability of survival (**Figures 7A, C**). The immune checkpoint and immune-activating genes (IDO1, CD274 (PD1), HAVCR2, PDCD1 (PD-L1), CTLA4, LAG3, CD8A, CXCL10, CXCL9, GZMA, GZMB, PRF1, IFNG, TBX2, and TNF) were selected as the target genes, and their differential expression in the ICI High and Low Scores groups was observed. Except for TBX2, all related genes exhibited significantly higher expression in the ICI Low Score group than in the High Score group (p >0.05) (**Figure 7B**). GSEA was conducted to identify the different functional pathways in the ICI High and Low Scores groups; 375 pathways were enriched in the ICI High Scores group and 429 in the ICI Low Scores group. We selected the top 5 related pathways for visualization: Taste Transduction, Calcium Signaling Pathway, Vascular Smooth Muscle Contraction, Neuroactive Ligand Receptor Interaction, and Vasopressin Regulated Water Reabsorption in the ICI High score group and Spliceosome, Proteasome, RNA Degradation, Nucleotide Excision Repair, and DNA Replication in the ICI Low scoring group (**Figure 7D**).

The current evidence suggests that tumor mutation burden (TMB) is an effective biomarker for immunotherapy of various tumors (24). Previous studies have shown that the TMB High group is more sensitive to ICIs for tumor patients with immunotherapy (25). Stratified analysis is an effective method to explore the relationship between a new model of tumor immunotherapy prognosis and TMB (26). According to the immune characteristics of TMB, we divided the MFS samples into the TMB High and TMB Low groups. The TMB High group had a significantly better OS (p = 0.027) (**Figure 7E**). Then, we began assessing the potential link between TMB and ICI scores. In the stratified analysis, the survival status of ICI Low groups (yellow and purple) was significantly higher than that of ICI High groups (blue and red) (p = 0.033), and the survival status of the TMB High groups (yellow and bule) was higher than that of the TMB Low groups (red and purple) (**Figure 7F**). No relationship was found between the ICI scores and TMB results.

## Discussion

Current evidence suggests that tumor immunotherapy is more dependent on the interaction between tumor cells and the tumor microenvironment (TME) than histological findings (27). Overwhelming evidence substantiates that the immune infiltration microenvironment can be harnessed to predict the prognosis of gastric, breast, and lung cancers (28–31). The identification of gene deletions in tumor samples is highly significant for tumor treatment. Interestingly, it has been shown that there are more gene deletions in sarcoma samples with low immune infiltration, while the samples with high immune infiltration exhibit stronger adaptability to the therapeutic effect (32). Similarly, recent studies have proposed that the polygenic immune risk score model based on immune cell infiltration in osteosarcoma is a reliable prognostic tool for osteosarcoma (33, 34). Nonetheless, few studies have hitherto been conducted on MFS. Importantly, our study can improve the current knowledge on the prognosis of MFS from the perspective of autoimmunity.

First, the immune cells infiltrated in 104 MFS samples from different databases were classified by unsupervised cluster analysis. The difference in prognostic information was analyzed based on the obtained ICI classification and the relationships among 24 immune infiltration characteristics (22 immune infiltration cells and immune score, stromal score) were explored. Multiple immune checkpoint-related genes were selected as immune checkpoints, and their differences in expression were analyzed based on immune infiltration typing. Subsequently, after obtaining DEGs related to immune infiltration, genotyping was carried out by unsupervised cluster analysis. Genotyping was used to analyze the differences between clinical prognosis and outcome of MFS samples; the consistency with ICI typing results was verified. Meanwhile, the characteristic genes related to immune infiltration were obtained using “Boruta” and “PCA.” Then, the ICI High and Low score groups were obtained by gene analysis of immune infiltration. For this step, GO enrichment analysis was used to explore the genes and protein functions related to the ICI High and Low score groups. At the same time, the differences in immune checkpoint genes were analyzed again by gene typing. The results were compared with the results of the first step to verify the stability and rationality of our ICI typing. Finally, the difference between immune checkpoint and immune activation-related genes was analyzed by the ICI score obtained during the second step, and GSEA enriched the top five signaling pathways between ICI high and low score groups. TMB is often considered as the number of tumor mutations (35). It is well-established that TMB could be used as an independent biomarker related to ICI in various solid tumors. Therefore, the feasibility of our MFS-related ICI prediction model was validated by comparing TMB with our established ICI score (36). Our results showed that the established ICI score had a definite guide value in predicting the prognosis of patients with MFS. However, the results were visualized in a Sankey diagram. It was found that the ICI score could not accurately predict the prognosis, which may be related to the differences in other cytokines, components, and localization points of TME during GO analysis of MFS. These results show that unidentified biological processes may affect the accuracy of our ICI prognosis model.

Interestingly, Chen et al. documented an unprecedented immunophenotypic typing in 2017 (37), including immune-inflamed (characterized by a large number of infiltrated immune cells, B cell activation, T regulatory cell participation, and T cell depletion; checkpoints inhibitors exert an effective antitumor effect), immune-excluded (characterized by a large number of immune cells in the stroma, with high immune and stromal scores, and few immune infiltrating cells overall; checkpoint inhibitors yield poor antitumor efficacy) and immune-desert tumors (characterized by few or no CD8 T cells in the whole sample, exhibiting immune tolerance or immune neglect; checkpoints inhibitors exert no effect). According to the results in **Figures 2D**, **5B**, ICI cluster A and gene cluster A showed the lowest expression of B and T cells, and immune infiltration levels were generally low; cluster C showed higher expression of the immune score and T cells, especially CD8 T cells. The difference in immune infiltration characteristics in our study was consistent with the three immune phenotypes, which further validated the rationality of our typing approach: an immune-inflamed tumor corresponded to cluster C (red), an immune-excluded tumor to cluster A (blue), and an immune-desert tumor to cluster B (yellow). At the same time, the high expression of immune checkpoint genes in cluster C emphasized the importance of selecting checkpoint inhibitors to achieve an antitumor effect. Our results were consistent with the literature (36, 38, 39), which proved that typing ICI and gene clusters could guide clinical immunotherapy of MFS. Our findings substantiate that the increase of immune infiltrating cells in the TME is a positive factor for the prognosis of the tumor. Therefore, immunotherapies that can improve the degree of immune cell infiltration in the TME are worth advocating. Interestingly, it has been reported that autologous dendritic cell immunotherapy could produce an active immune response in tumors, but reliable biomarkers are warranted to guide the treatment plan (10).

According to the GSEA results, the high-scoring group of tumor samples in ICI was significantly enriched in the activation and proliferation of immune cells. In a study on tumor dichotomy (hot tumor and cold tumor), Li et al. documented that immunotherapy yielded a better effect on hot tumors. In mice experiments, the six-month survival rates of the hot and cold tumor groups were 76.9 and 0.5%, respectively (40). The overlap in characteristics of hot tumors suggested that the prognosis of immunotherapy accounted for the better prognosis in ICI cluster C (41). The difference between the ICI score and the TMB score was significant (P = 0.033), which corroborated that the ICI score was a new valuable independent score.

However, we could not further validate the ICI score given the lack of MFS samples, which were from public databases or case collections of our research group. Our ICI score only evaluated the prognosis of MFS from the perspective of immune infiltration without considering other complex mechanisms in TME. At the same time, the difference in some results (especially between immune excluded and immune inflamed) was not significant due to the sample size limitation.

## Conclusion

Our study comprehensively analyzed the ICI characteristics of MFS, established the effectiveness of ICI typing and gene typing, predicted the prognosis of MFS samples through ICI scores, and evaluated the therapeutic effect of MFS under ICI typing along with differences in immune checkpoint-related genes, which could assist physicians in developing individualized immunotherapy schemes and prognosis prediction.

## Data Availability Statement

The datasets presented in this study can be found in online repositories. The names of the repository/repositories and accession number(s) can be found in the article/supplementary material.

## Author Contributions

Z-YZ, Z-YC, HL, and TX designed and implemented the research. Z-YZ, Z-YC, and BY collated and analyzed the data. BX, L-YL, YX, A-YL, P-XW, CX CL, and H-QY provided technical support. Z-YC and HL provided the language polishing for this article. Z-YZ wrote the manuscript. Z-YZ and HL revised the manuscript. All authors listed have made a substantial, direct, and intellectual contribution to the work and approved it for publication.

## Funding

The study was supported by the Research Project of Hunan Health Commission (grant number 202204073071).

## Conflict of Interest

The authors declare that the research was conducted in the absence of any commercial or financial relationships that could be construed as a potential conflict of interest.

## Publisher’s Note

All claims expressed in this article are solely those of the authors and do not necessarily represent those of their affiliated organizations, or those of the publisher, the editors and the reviewers. Any product that may be evaluated in this article, or claim that may be made by its manufacturer, is not guaranteed or endorsed by the publisher.

## Abbreviations

MFS, myxofibrosarcoma;ICI, immune cell infiltration; GO, Gene Ontology; GSEA, Gene Set Enrichment Analysis; TME, tumor microenvironment; ICIs, immune checkpoint inhibitors; ASPS, alveolar soft part sarcoma; UPS, undifferentiated pleomorphic sarcoma; PP, pseudo-progression; HP, hyper-progression; mrcc, metabolic renal cell carcinoma; HCC, hepatocellular carcinoma; tkis, tyrosine kinase inhibitors; TCGA, The Cancer Genome Atlas; GEO, Gene Expression Omnibus database; FPKM, fragments per kilobase per million; DEGs, differentially expressed genes; TMB, tumor mutational burden; OS, overall survival time; BP, biological process; CC, cellular component; MF, molecular function.

## References

[1] Sambri A Spinnato P Bazzocchi A Tuzzato GM Donati D Bianchi G. Does pre-operative MRI predict the risk of local recurrence in primary myxofibrosarcoma of the extremities? Asia Pac J Clin Oncol (2019) 15:e181–6. doi: 10.1111/ajco.13161 31111597

[2] Rothenberger C Jakob L Betke M French L Bartsch H Knosel T. Myxofibrosarcoma. Hautarzt (2020) 71:30–1. doi: 10.1007/s00105-020-04647-7 32974710

[3] Anderson WJ Doyle LA. Updates from the 2020 world health organization classification of soft tissue and bone tumours. Histopathology (2021) 78:644–57. doi: 10.1111/his.14265 33438273

[4] Palmerini E Righi A Staals EL. Rare primary malignant bone sarcomas. Cancers (Basel) (2020) 124. doi: 10.3390/cancers12113092 PMC7690832 33114111

[5] Murphey MD. World health organization classification of bone and soft tissue tumors: modifications and implications for radiologists. Semin Musculoskelet Radiol (2007) 11:201–14. doi: 10.1055/s-2008-1038310 18260031

[6] Widemann BC Italiano A. Biology and management of undifferentiated pleomorphic sarcoma, myxofibrosarcoma, and malignant peripheral nerve sheath tumors: State of the art and perspectives. J Clin Oncol (2018) 36:160–7. doi: 10.1200/JCO.2017.75.3467 PMC5759316 29220302

[7] Zambo I Veselý K. [WHO classification of tumours of soft tissue and bone 2013: the main changes compared to the 3rd edition]. Cesk Patol (2014) 50:64–70. 24758500

[8] Roland CL Wang WL Lazar AJ Torres KE. Myxofibrosarcoma. Surg Oncol Clin N Am (2016) 25:775–88. doi: 10.1016/j.soc.2016.05.008 27591498

[9] Zhang Y Zhang Z. The history and advances in cancer immunotherapy: understanding the characteristics of tumor-infiltrating immune cells and their therapeutic implications. Cell Mol Immunol (2020) 17:807–21. doi: 10.1038/s41423-020-0488-6 PMC7395159 32612154

[10] Sokratous G Polyzoidis S Ashkan K. Immune infiltration of tumor microenvironment following immunotherapy for glioblastoma multiforme. Hum Vaccin Immunother (2017) 13:2575–82. doi: 10.1080/21645515.2017.1303582 PMC5703406 28362548

[11] Keenan TE Tolaney SM. Role of immunotherapy in triple-negative breast cancer. J Natl Compr Canc Netw (2020) 18:479–89. doi: 10.6004/jnccn.2020.7554 32259782

[12] Iams WT Porter J Horn L. Immunotherapeutic approaches for small-cell lung cancer. Nat Rev Clin Oncol (2020) 17:300–12. doi: 10.1038/s41571-019-0316-z PMC7212527 32055013

[13] Huang A Yang XR Chung WY Dennison AR Zhou J. Targeted therapy for hepatocellular carcinoma. Signal Transduct Target Ther (2020) 5:146. doi: 10.1038/s41392-020-00264-x 32782275 PMC7419547

[14] Duma N Santana-Davila R Molina JR. Non-small cell lung cancer: Epidemiology, screening, diagnosis, and treatment. Mayo Clin Proc (2019) 94:1623–40. doi: 10.1016/j.mayocp.2019.01.013 31378236

[15] Kwapisz D. Pembrolizumab and atezolizumab in triple-negative breast cancer. Cancer Immunol Immunother (2021) 70:607–17. doi: 10.1007/s00262-020-02736-z PMC10992894 33015734

[16] Jia XH Geng L-Y Jiang P-P Xu H Nan K-J Yao Y Jiang L-L. The biomarkers related to immune related adverse events caused by immune checkpoint inhibitors. J Exp Clin Cancer Res (2020) 39:284. doi: 10.1186/s13046-020-01749-x 33317597 PMC7734811

[17] Keung EZ Lazar AJ Torres KE Wang W-L Cormier JN Guadagnolo BA. Phase II study of neoadjuvant checkpoint blockade in patients with surgically resectable undifferentiated pleomorphic sarcoma and dedifferentiated liposarcoma. BMC Cancer (2018) 18:913. doi: 10.1186/s12885-018-4829-0 30249211 PMC6154892

[18] Petitprez F Reyniès A Keung EZ Chen TW-W Sun C-M Calderaro J Jeng Y-M. B cells are associated with survival and immunotherapy response in sarcoma. Nature (2020) 577:556–60. doi: 10.1038/s41586-019-1906-8 31942077

[19] Kim SK Kim JH Kim SH Lee YH Han JW Baek W. PD-L1 tumour expression is predictive of pazopanib response in soft tissue sarcoma. BMC Cancer (2021) 21:336. doi: 10.1186/s12885-021-08069-z 33789622 PMC8011221

[20] You Y Guo X Zhuang R Zhang C Wang Z Shen F. Activity of PD-1 inhibitor combined with anti-angiogenic therapy in advanced sarcoma: A single-center retrospective analysis. Front Mol Biosci (2021) 8:747650. doi: 10.3389/fmolb.2021.747650 34869583 PMC8635153

[21] de Miguel M Calvo E. Clinical challenges of immune checkpoint inhibitors. Cancer Cell (2020) 38:326–33. doi: 10.1016/j.ccell.2020.07.004 32750319

[22] Rizzo A Mollica V Santoni M Ricci AD Rosellini M Marchetti A. Impact of clinicopathological features on survival in patients treated with first-line immune checkpoint inhibitors plus tyrosine kinase inhibitors for renal cell carcinoma: A meta-analysis of randomized clinical trials. Eur Urol Focus (2021) EUF-1077:17. doi: 10.1016/j.euf.2021.03.001 33714725

[23] Rizzo A Ricci AD. PD-L1, TMB, and other potential predictors of response to immunotherapy for hepatocellular carcinoma: how can they assist drug clinical trials? Expert Opin Investig Drugs (2022) 31:415–23. doi: 10.1080/13543784.2021.1972969 34429006

[24] Chan TA Yarchoan M Jaffee E Swanton C Quezada SA Stenzinger A. Development of tumor mutation burden as an immunotherapy biomarker: utility for the oncology clinic. Ann Oncol (2019) 30:44–56. doi: 10.1093/annonc/mdy495 30395155 PMC6336005

[25] Cristescu R Mogg R Ayers M Albright A Murphy E Yearley J. Pan-tumor genomic biomarkers for PD-1 checkpoint blockade-based immunotherapy. Science (2018) 3622. doi: 10.1126/science.aar3593 PMC6718162 30309915

[26] Liu L Bai X Wang J Tang X-R Wu D-H Du S-S. Combination of TMB and CNA stratifies prognostic and predictive responses to immunotherapy across metastatic cancer. Clin Cancer Res (2019) 25:7413–23. doi: 10.1158/1078-0432.CCR-19-0558 31515453

[27] Mittra A Takebe N Florou V Chen AP Naqash AR. The emerging landscape of immune checkpoint inhibitor based clinical trials in adults with advanced rare tumors. Hum Vaccin Immunother (2021) 17:1935–9. doi: 10.1080/21645515.2020.1854604 PMC8189105 33325769

[28] Li B Zhang B Wang X Zeng Z Huang Z Zhang L. Expression signature, prognosis value, and immune characteristics of siglec-15 identified by pan-cancer analysis. Oncoimmunology (2020) 9:1807291. doi: 10.1080/2162402X.2020.1807291 32939323 PMC7480813

[29] Zhang B Wu Q Li B Wang D Wang L Zhou YL. m(6)A regulator-mediated methylation modification patterns and tumor microenvironment infiltration characterization in gastric cancer. Mol Cancer (2020) 19:53. doi: 10.1186/s12943-020-01170-0 32164750 PMC7066851

[30] Wang S Zhang Q Yu C Cao Y Zuo Y Yang L. Immune cell infiltration-based signature for prognosis and immunogenomic analysis in breast cancer. Brief Bioinform (2021) 22:2020–31. doi: 10.1093/bib/bbaa026 32141494

[31] Sun J Zhang Z Bao S Yan C Hou P Wu N. Identification of tumor immune infiltration-associated lncRNAs for improving prognosis and immunotherapy response of patients with non-small cell lung cancer. J Immunother Cancer (2020) 8:1–10. doi: 10.1136/jitc-2019-000110 PMC7057423 32041817

[32] Wu CC Beird HC Livingston JA Advani S Mitra A Cao S. Immuno-genomic landscape of osteosarcoma. Nat Commun (2020) 11:1008. doi: 10.1038/s41467-020-14646-w 32081846 PMC7035358

[33] Fan L Ru J Liu T Ma C. Identification of a novel prognostic gene signature from the immune cell infiltration landscape of osteosarcoma. Front Cell Dev Biol (2021) 9:718624. doi: 10.3389/fcell.2021.718624 34552929 PMC8450587

[34] Chen Y Zhao B Wang X. Tumor infiltrating immune cells (TIICs) as a biomarker for prognosis benefits in patients with osteosarcoma. BMC Cancer (2020) 20:1022. doi: 10.1186/s12885-020-07536-3 33087099 PMC7579940

[35] Cheng X Wang X Nie K Cheng L Zhang Z Hu Y. Systematic pan-cancer analysis identifies TREM2 as an immunological and prognostic biomarker. Front Immunol (2021) 12:646523. doi: 10.3389/fimmu.2021.646523 33679809 PMC7925850

[36] Zhang X Shi M Chen T Zhang B. Characterization of the immune cell infiltration landscape in head and neck squamous cell carcinoma to aid immunotherapy. Mol Ther Nucleic Acids (2020) 22:298–309. doi: 10.1016/j.omtn.2020.08.030 33230435 PMC7522342

[37] Chen DS Mellman I. Elements of cancer immunity and the cancer-immune set point. Nature (2017) 541:321–30. doi: 10.1038/nature21349 28102259

[38] Park S Ock C-Y Kim H Pereira S Park S Ma M. Artificial intelligence-powered spatial analysis of tumor-infiltrating lymphocytes as complementary biomarker for immune checkpoint inhibition in non-Small-Cell lung cancer. J Clin Oncol (2022) 40(17):JCO2102010. doi: 10.1200/JCO.21.02010 PMC9177249 35271299

[39] Xiao Y Ma D Zhao S Suo C Shi J Xue M-Z. Multi-omics profiling reveals distinct microenvironment characterization and suggests immune escape mechanisms of triple-negative breast cancer. Clin Cancer Res (2019) 25:5002–14. doi: 10.1158/1078-0432.CCR-18-3524 30837276

[40] Li J Byrne KT Yan F Yamazoe T Chen Z Baslan T. Tumor cell-intrinsic factors underlie heterogeneity of immune cell infiltration and response to immunotherapy. Immunity (2018) 49:178–193.e177. doi: 10.1016/j.immuni.2018.06.006 29958801 PMC6707727

[41] Galon J Bruni D. Approaches to treat immune hot, altered and cold tumours with combination immunotherapies. Nat Rev Drug Discov (2019) 18:197–218. doi: 10.1038/s41573-018-0007-y 30610226

